# Trends in mortality from external causes in the Republic of Seychelles between 1989 and 2018

**DOI:** 10.1038/s41598-020-79228-8

**Published:** 2020-12-17

**Authors:** Anne Abio, Pascal Bovet, Joachim Didon, Till Bärnighausen, Masood Ali Shaikh, Jussi P. Posti, Michael Lowery Wilson

**Affiliations:** 1 Injury Epidemiology and Prevention Research Group, Division of Clinical Neurosciences, Turku Brain Injury Centre, Turku University Hospital and University of Turku, Turku, Finland; 2 University Center for Primary Care and Public Health (Unisanté), Lausanne, Switzerland; 3 Ministry of Health, Victoria, Republic of Seychelles; 4 Heidelberg Institute of Global Health, University of Heidelberg, Heidelberg, Germany; 5 Division of Clinical Neurosciences, Department of Neurosurgery and Turku Brain Injury Centre, Turku University Hospital and University of Turku, Turku, Finland

**Keywords:** Trauma, Epidemiology, Public health

## Abstract

Data on injury-related mortality are scarce in the African region. Mortality from external causes in the Seychelles was assessed, where all deaths are medically certified and the population is regularly enumerated. The four fields for underlying causes of death recorded were reviewed in the national vital statistics register. The age-standardised mortality rates were estimated (per 100,000 person-years) from external causes in 1989–1998, 1999–2008, and 2009–2018. Mortality rates per 100,000 person-years from external causes were 4–5 times higher among males than females, and decreased among males over the three 10-year periods (127.5, 101.4, 97.1) but not among females (26.9, 23.1, 26.9). The contribution of external causes to total mortality did not change markedly over time (males 11.6%, females 4.3% in 1989–2018). Apart from external deaths from undetermined causes (males 14.6, females 2.4) and “other unintentional injuries” (males 14.1, females 8.0), the leading external causes of death in 2009–2018 were drowning (25.9), road traffic injuries (18.0) and suicide (10.4) among males; and road traffic injuries (4.6), drowning (3.4) and poisoning (2.6) among females. Mortality from broad categories of external causes did not change consistently over time but rates of road traffic injuries increased among males. External causes contributed approximately 1 in 10 deaths among males and 1 in 20 among females, with no marked change in cause-specific rates over time, except for road traffic injuries. These findings emphasise the need for programs and policies in various sectors to address this large, but mostly avoidable health burden.

## Introduction

Global mortality due to external causes was estimated at 73 per 100,000 population in 2012^1^. Mortality rates per year and per 100,000 inhabitants varied by region and were, for example, 99 in South East Asia, 62 in the Americas and 49 in Europe in 2012^1^. By contrast, it was estimated, despite the lack of country-wide data on both cause-specific mortality and population distribution in most countries in the region, that the age-standardised mortality from external causes was as high as 116 per 100,000 inhabitants in 2012 in Africa^1^, with road traffic injuries often contributing the largest burden^2,3^. According to the World Health Organisation (WHO), and based on previously reported data from the same database used in this study, the age-standardised mortality rates from external causes in the Seychelles in 2008 were 81 and 17 per 100,000 inhabitants among males and females, respectively^4^, with road traffic related mortality being 8.6 per 100,000 inhabitants in 2013^2,3^.

Broad categories of external causes of death can be defined based on the nature of the causes of injuries, and include road traffic injuries, drowning, suicide, poisoning, homicide, falls, other unintentional injuries, other intentional injuries and those with undetermined intent^5^. Globally, the leading five external causes of death include road traffic injuries, other unintentional injuries, suicide, homicide and falls^6,7^. Worldwide, road traffic injuries account for roughly a quarter of all deaths from external causes, with, for example, an estimated 1.25 million deaths occurring in 2013, at a rate of 24.1 deaths per 100,000 inhabitants^2,3,6^. Studies conducted in Ethiopia, Kenya and Guinea showed that road traffic injuries, homicide and drowning were usually among the three leading causes of mortality, accounting for 19–46% of from total mortality due to external causes^8–11^.

Within the African region, Seychelles had the highest Sustainable Development Goals Index score at 71 suggesting it was leading on the progress towards achieving the goals^12–14^. Epidemiological research and surveillance on external causes of death remain under-addressed compared to other health issues in Africa^15,16^. A reason is that data on external causes of death are scarce and incomplete in most countries in the region, is largely because causes of death are not registered at the whole population level. Furthermore, the age distribution of the population is not available at the national level in some countries within the region. By contrast, all deaths have been medically certified and registered (national vital statistics), and the population fully enumerated through regular censuses (with data thereafter are updated by the civil authorities) for more than three decades in the Seychelles, a small island state in the Indian Ocean off the east coast of Kenya^17^. The availability of complete data on both the cause-specific mortality and the population distribution in a country in the African region represents a valuable opportunity to describe secular trends in mortality due to external causes in this region. This allowed us to review, and recode, all certificates in the considered 30-year study period, irrespective of which ICD or other classification was used at the time of death. This study therefore aimed at examining age-standardised mortality rates from broad causes of external deaths over the 30 past years (1989 to 2018).

## Methodology

### Data and population

**Figure 1:**
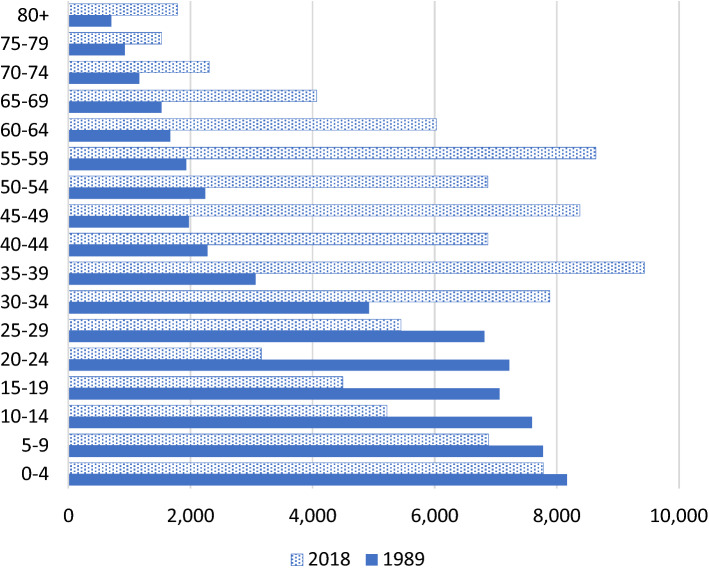
Age structure of the population of Seychelles in 1989 and 2018.

In Seychelles, the entire population has been regularly enumerated for the last five decades at approximately ten year intervals using national census information. The data are subsequently regularly updated by the civil authorities. In 2019, Seychelles had a population of 97,625^18^. There has been a rapid demographic transition between 1989 and 2018, with a population that is rapidly increasing and aging^19^ (Fig. 1). In addition to decreasing mortality and birth rates, there have been substantial migration movements (e.g. emigration of young Seychellois to pursue studies and training abroad and the immigration of foreign workers).

For more than 3 decades, the causes of all deaths have been certified by a physician using death certificates which included four fields for the cause(s) of death (underlying, immediate, intermediate and contributing causes of death), and the data was compiled within an electronic register^17^. This study used data that was available between 1989 and 2018.

For the purpose of this study, the four fields for the causes of death were manually reviewed whenever an external cause of death was mentioned in any of these four fields. Of note, the register of vital statistics includes the textual description of the underlying and associated causes of deaths as written by the physician who completed the death certificate at the time of a person’s death. Therefore, a death was considered to be due to an “external cause” if an external cause was mentioned in any of the four fields. Where the information in one of the 4 fields corresponded explicitly to an external cause of death, with a related code mentioned along the WHO International Classification of Diseases, 10th revision (ICD-10), these codes were used without modification. Where ambiguity existed about the cause of death (because the text of the cause of death was unclear, or the text in two or more of the fields for underlying causes of death appeared contradictory, etc.), two authors (AA and MLW) made a joint decision as to the final classification. Hence, the coding process of all external causes of death considered in this study was consistent with the ICD-10 classification. The following ICD-10 codes for external causes were considered: S00–S99, T00–T98, U01–U03, V01–V99, W00–W99, X00–X84, X85–Y05, Y08–Y09, Y10–Y36, and Y85–Y89. In view of the limited number of cases in our database, these diagnostic categories were expanded into broader categories as follows: drowning, road traffic injuries, suicide, homicide, poisoning, falls, fire related injuries, other unintentional injuries, and injuries with undetermined intent. The category “other unintentional injuries” included, mainly, deaths related to limb fractures among the elderly, electrocution, shark attacks, helicopter crashes, injury by an aircraft propeller, ship injuries, rock accidents, struck by falling objects, lightning, other asphyxiation, and injuries due to epileptic fits. External causes with “undetermined intent” mainly included cases where the cause of the injury was not specified and thus, the exact external cause of death was challenging to ascertain from the database. Examples of these included skull fractures, head injuries, multiple injuries, penetrating wounds and trauma where a specific cause was not mentioned. It is possible that some cases within the ‘undetermined intent’ category could have been homicides or femicides that were inadequately documented. Homicide cases included mainly assault, stab wounds and gunshot wounds. Age at death (data were available in rounded number years) and sex were also collected from the death registry. No other data (in particular names of persons or names of place of death, or name of doctor who completed the death certificate) was included in the database used for the study.

### Statistical analysis

Age-standardised mortality rates were computed for each defined category of external causes of death by directly standardising the mortality rates to the age distribution of the WHO standard population^20^. Deaths and population sizes were pooled during three ten-year periods (1989–1998, 1998–2008, 2009–2018) due to the small numbers of certain external causes of death, given the small population in Seychelles. Of note, the fact that mortality data are based on all deaths occurring in the entire population limits the need for using statistical tests, which are suitable for data based on random sub-samples of a population. However, considering that deaths occurring on any particular year may be considered as a sub-sample from a broader population with random variation over time, significant differences in mortality rates between the three ten-year periods were tested using negative binomial regression models after adjusting for age. The data analysis was done using Stata 11 (StataCorp, TX, USA). Authorisation to use these data was obtained from the Ministry of Health, Republic of Seychelles. Furthermore, the Ethical Commission of the University of Heidelberg Medical Faculty, upon assessment of the study protocol, declared that the study did not require additional ethical approval given the anonymous nature of the data, the use of aggregate estimates, and the retrospective design of the study.

## Results

**Figure 2:**
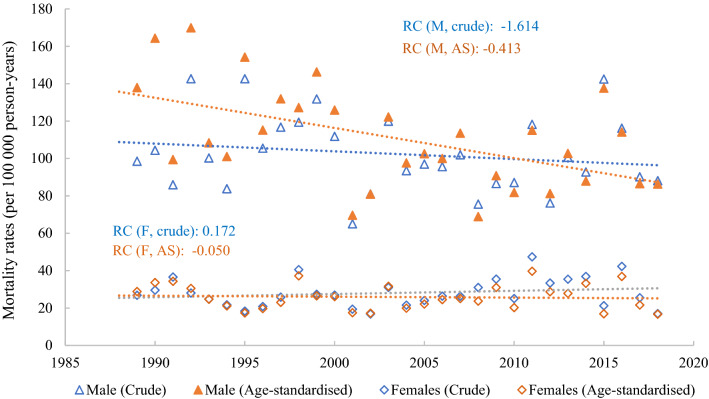
Crude and age-standardised mortality rates from external causes between 1989 and 2018 in males and females with linear trends (RC: linear regression coefficient; M: males; F: females; AS: age-standardised).

From 1989 to 2018, a total of 18,961 deaths occurred in the Seychelles. During the same period, 1262 deaths attributed to external causes were reported (Table 1). Figure 2 shows the crude and age-standardised mortality rates for all causes and for external causes while Table 2 shows the rates by category for the entire period. The age-adjusted all-cause mortality rates (per 100,000 person-years) were twice as large among males (1092.9) than among females (545.8). The age-adjusted mortality rates from external causes were more than 6 times larger among males (25.9) than among females (3.4). The proportion of external causes of death to all causes of death was 11.6% among males and 4.3% among females. Beyond the mortality rates from “undetermined causes” (males 14.6, females 2.4) and those from “other unintentional injuries” (males 14.1, females 8.0), the highest mortality rates from external causes were drowning (25.9), road traffic injuries (18.0) and suicide (10.4) in males, and road traffic injuries (4.6), drowning (3.4) and poisoning (2.6) among females.

**Table 1:** Descriptive statistics.

Years	1989–2018		Numbers (%)					
			1989–1998		1999–2008		2009–2018	
Category	M	F	M	F	M	F	M	F
Drowning	310 (88.1)	42 (11.9)	105 (90.5)	11 (9.5)	91 (88.4)	12 (11.6)	114 (85.7)	19 (14.3)
Road traffic injuries	225 (79.8)	57 (20.2)	58 (80.6)	14 (19.4)	73 (76.8)	22 (23.2)	94 (81.7)	21 (18.3)
Suicide	129 (92.8)	10 (7.2)	35 (97.2)	01 (2.8)	49 (92.5)	04 (7.5)	45 (90.0)	05 (10.0)
Homicide	89 (76.7)	27 (23.3)	23 (82.1)	05 (17.9)	44 (81.5)	10 (18.5)	22 (64.7)	12 (35.3)
Poisoning	89 (73.6)	32 (26.4)	34 (75.6)	11 (24.4)	11 (68.7)	05 (31.3)	44 (73.3)	16 (26.7)
Falls	56 (87.5)	08 (12.5)	10 (83.3)	02 (16.7)	18 (81.8)	04 (18.2)	28 (93.3)	02 (6.7)
Fire related burns	42 (77.8)	12 (22.2)	08 (66.7)	04 (33.3)	14 (87.5)	02 (12.5)	20 (76.9)	06 (23.1)
Other unintentional injuries	150 (53.8)	129 (46.2)	50 (56.8)	38 (43.2)	49 (57.0)	37 (43.0)	51 (48.6)	54 (51.4)
External causes, undetermined	172 (84.3)	32 (15.7)	77 (84.6)	14 (15.4)	55 (87.3)	08 (12.7)	40 (80.0)	10 (20.0)
All external causes	1262 (78.3)	349 (21.7)	400 (80.0)	100 (20.0)	404 (79.5)	104 (20.5)	458 (76.0)	145 (24.0)
**By age groups (all external causes)**								
0–19	120 (72.3)	46 (27.7)	55 (72.4)	21 (27.6)	37 (69.8)	16 (30.2)	28 (75.7)	09 (24.3)
20–39	507 (85.2)	88 (14.8)	163 (88.1)	22 (11.9)	168 (86.6)	26 (13.4)	176 (81.5)	40 (18.5)
40–59	380 (87.6)	54 (12.4)	100 (90.9)	10 (9.1)	126 (87.5)	18 (12.5)	154 (85.6)	26 (14.4)
60+	255 (61.3)	161 (38.7)	82 (63.6)	47 (36.4)	73 (62.4)	44 (37.6)	100 (58.8)	70 (41.2)

*M* males; *F* females.

**Table 2:** Crude and age-standardised mortality rates by age and sex in 1989–2018.

	Crude mortality rate		Age-standardised mortality rate		95% confidence interval of the age-standardised rates	
	M	F	M	F	M	F
All-cause mortality	879.8	652.6	1092.9	545.8	1072.9, 1113.0	533.9, 557.7
All external causes	102.0	28.2	105.5	25.4	99.6, 111.4	22.7, 28.1
Drowning	25.0	3.4	25.9	3.4	23.0, 28.9	2.4, 4.5
Road traffic injuries	18.2	4.6	18.0	4.6	15.7, 20.4	3.4, 5.8
Suicide	10.4	0.8	10.4	0.8	8.6, 12.2	0.3, 1.3
Homicide	7.2	2.2	6.8	2.1	5.4, 8.3	1.3, 3.0
Poisoning	7.2	2.6	7.0	2.6	5.5, 8.5	1.7, 3.4
Falls	4.5	0.6	4.9	0.6	3.6, 6.2	0.2, 0.9
Fire related burns	3.4	1.0	3.6	0.9	2.5, 4.7	0.4, 1.5
Other unintentional injuries	12.1	10.4	14.1	8.0	11.8, 16.5	6.6, 9.4
External causes, undetermined	13.9	2.6	14.6	2.4	12.4, 16.9	1.5, 3.2

*M* males; *F* females.

Table 3 shows the distribution of the population, the age-adjusted all-cause mortality rates, and the mortality rates due to external causes according to sex and age. The all-cause mortality rates increased markedly with age. The all-cause mortality rates did not change significantly over time among the younger male age categories, but appeared to increase among females. On the other hand, it decreased substantially among the older age categories, so that the overall total mortality rates decreased from 1989–1998 to 2009–2018 (from 1089.0 to 978.0 among males and from 606.6 to 504.3 among females). The mortality rates for external causes tended to decrease from 1989–1998 to 2009–2018 in most age categories among males (from 127.6 to 95.1) but no consistent change was observed among females (from 28.1 to 29.3). External causes of deaths contributed 12.1%, 11.5% and 11.3% of all deaths among males in 1989–1998, 1999–2008, 2009–2018, respectively, and 4.3%, 3.8% and 4.8% among females, suggesting that these proportions did not change substantially according to the considered time periods.

**Table 3:** Distribution of the population, age-adjusted all-cause mortality, and mortality due to external causes according to sex and age in 1989–1998, 1999–2008 and 2009–2018.

	Males				Females			
	1989–1998	1999–2008	2009–2018	P trend	1989–1998	1999–2008	2009–2018	P trend
**All causes**								
Age standardised mortality								
0–19	46.6	40.8	46.7		30.7	33.4	37.2	
20–39	83.9	74.3	78.7		27.7	27.9	42.2	
40–59	297.8	221.7	198.9		100.1	87.4	80.0	
60+	836.3	728.0	571.2		514.1	488.3	420.8	
All ages	1329.9	1089.0	978.0	0.05	606.6	556.8	504.3	0.01
(95% CI)	(1286, 1374)	(1054, 1124)	(948, 1008)		(582, 632)	(535, 578)	(486, 522)	
**External causes**								
Age standardised mortality								
0–19	12.6	9.0	7.4		4.9	4.0	2.5	
20–39	39.7	34.7	36.5		5.5	5.6	9.2	
40–59	40.1	31.9	26.9		4.2	4.9	4.5	
60+	34.5	25.1	23.8		13.2	10.1	13.0	
All ages	127.6	101.2	95.1	0.25	28.1	24.9	29.3	0.32
(95% CI)	(115, 141)	(91, 112)	(88, 106)		(22, 32)	(19, 28)	(22, 32)	
**Proportion of external causes from total mortality (%)**								
	10.1	9.5	10.7		4.2	3.9	5.1	

All mortality rates are expressed per 100,000 person-years.

Table 4 shows the age-standardised mortality rates (per 100,000 person-years) for the defined broad categories of external causes according to sex and age in 1989–1998, 1999–2008 and 2009–2018. Data among females were based on small numbers and the secular trends could therefore not be established accurately; yet, no consistent trend over time seemed to appear. Among males, no trend over time reached statistical significance. However, mortality rates for drowning seemed to decrease substantially between 1989–1998 and 2009–2018, especially among persons aged less than 40 years (by almost a third). Mortality rates for road traffic injury deaths seemed to increase among persons aged above 20 years and decrease among persons aged less than 20 years. Mortality rates for poisoning (which often relates to suicide in the Seychelles context) did not seem to change over time, with more than half of all cases occurring at the ages 20–39 years. No clear trend over time was found for mortality due to homicide with more than half of all cases occurring among persons aged 20–39 years. Mortality rates due to falls or fire-related burns were very low and trends over time could not be assessed reliably. Mortality rates due to other unintentional causes decreased from 17.7 in 1989–1998 to 12.6 in 2009–2018. Rates from other unintentional injuries were fairly high among both males and females, and decreased slightly (17.7, 13.3 and 12.6 in males in the three time periods) and in females (9.2, 6.9, 7.9). A sub-analysis showed that mortality rates due to “stabs or penetrating wounds”, which often relate to fights and homicides, were 3.7, 5.4 and 2.0 for males, and 0.2, 1.5 and 0.6 for females in the three ten-year periods. Most of these deaths occurred among persons aged 20 to 34 years. Finally, mortality rates from undetermined external causes decreased from 25.2 in 1989–1998 to 8.0 in 2009–2018, which may partly reflect better cause of death assessment in death certificates over time.

**Table 4:** Age-standardised mortality from broad categories of external causes according to sex and age in 1989–1998, 1999–2008 and 2009–2018.

	Males				Females			
	1989–1998	1999–2008	2009–2018	p trend	1989–1998	1999–2008	2009–2018	p trend
**Drowning**								
Mortality rate	33.7	23.6	23.7	0.36	3.0	3.1	3.9	0.41
(95% CI)	(27.1; 40.4)	(18.6; 28.6)	(19.3; 28.1)		(1.2; 4.9)	(1.3; 4.9)	(2.1; 5.6)	
0–19 (%)	14	9	6		36	25	16	
20–39 (%)	41	42	38		27	33	26	
40–59 (%)	25	31	34		18	25	42	
60+ (%)	21	18	22		18	17	16	
**Road traffic injuries**								
Mortality rate	16.6	17.3	20.5	0.10	4.5	5.3	4.5	0.73
(95% CI)	(12.1; 21.0)	(13.2; 21.3)	(16.3; 24.8)		(2.1; 7.0)	(3.0; 7.5)	(2.5; 6.5)	
0–19 (%)	29	15	14		21	32	14	
20–39 (%)	45	56	48		21	32	38	
40–59 (%)	19	22	26		43	23	19	
60+ (%)	7	7	13		14	14	29	
**Suicide**								
Mortality rate	10.9	11.8	9.0	0.93	0.3	1.1	1.1	0.22
(95% CI)	(7.1; 14.6)	(8.4; 15.1)	(6.3; 11.6)		(< 0.01; 1.0)	(< 0.01; 2.2)	(0.1; 2.2)	
0–19 (%)	6	4	2		0	0	20	
20–39 (%)	54	41	47		100	50	40	
40–59 (%)	31	45	36		0	25	40	
60 (%)	9	10	16		0	25	0	
**Poisoning**								
Mortality rate	11.0	2.4	8.9	0.63	3.0	1.2	3.5	0.63
(95% CI)	(7.1; 14.8)	(1.0; 3.7)	(6.2; 11.6)		(1.2; 4.8)	(0.1; 2.3)	(1.7; 5.2)	
0–19 (%)	3	18	0		27	20	0	
20–39 (%)	53	64	64		45	40	75	
40–59 (%)	26	18	30		0	20	19	
60 (%)	18	0	7		27	20	6	
**Homicide**								
Mortality rate	6.2	10.0	4.4	0.34	1.4	2.3	2.7	0.27
(95% CI)	(3.6; 8.8)	(7.0; 13.0)	(2.5; 6.2)		(0.1; 2.8)	(0.9; 3.8)	(1.1; 4.2)	
0–19 (%)	9	5	5		40	20	0	
20–39 (%)	70	66	50		40	30	58	
40–59 (%)	17	23	41		20	30	33	
60 (%)	4	7	5		0	20	8	
**Falls**								
Mortality rate	3.8	4.9	5.6	0.02	0.5	0.8	0.3	0.76
(95% CI)	(1.4; 6.2)	(2.6; 7.2)	(3.5; 7.8)		(< 0.01; 1.1)	(< 0.01; 1.7)	(< 0.01; 0.8)	
0–19 (%)	0	17	0		50	0	0	
20–39 (%)	20	17	32		50	25	0	
40–59 (%)	20	50	50		0	0	0	
60+ (%)	60	17	18		0	75	100	
**Fire related burns**								
Mortality rate	2.4	3.5	4.3	0.09	1.0	0.5	1.3	0.73
(95% CI)	(0.7; 4.2)	(1.6; 5.4)	(2.4; 6.3)		(< 0.01; 2.0)	(< 0.01; 1.2)	(0.2; 2.4)	
0–19 (%)	38	0	5		0	50	17	
20–39 (%)	25	43	35		50	0	50	
40–59 (%)	25	36	30		0	0	17	
60 (%)	13	21	30		50	50	17	
**Other unintentional injuries**^**a**^								
Mortality rate	17.7	13.3	12.6	0.33	9.2	6.9	7.9	0.60
(95% CI)	(12.6; 22.8)	(9.4; 17.1)	(9.0; 16.1)		(6.2; 12.1)	(4.6; 9.1)	(5.8; 10.1)	
0–19 (%)	14	6	4		11	3	0	
20–39 (%)	22	29	27		8	8	6	
40–59 (%)	20	24	16		0	8	4	
60+ (%)	44	41	53		82	81	91	
**External causes, undetermined**								
Mortality rate	25.2	14.8	8.0	< 0.001	3.9	1.9	1.7	0.14
(95% CI)	(19.3; 31.1)	(10.8; 18.8)	(5.5; 10.6)		(1.8; 5.9)	(0.5; 3.2)	(0.6; 2.8)	
0–19 (%)	10	4	3		29	13	10	
20–39 (%)	39	25	25		14	50	0	
40–59 (%)	27	42	53		7	25	20	
60+ (%)	23	29	20		50	13	70	

^a^E.g. limb fractures among the elderly, electrocution, shark attacks, hit by aircraft propeller, helicopter crashes, ship injuries, rock accidents, struck by falling objects, lightning, other asphyxiation, and injuries due to epileptic seizures.

## Discussion

The age-standardised rates for external causes of death during the three periods under study were 127.5, 101.4, and 97.1 among males; and 26.9, 23.1, and 26.9 among females, respectively. These rates closely reflect prior country-level estimates of 111 (males) and 26 (females) in 1989–1991 and 79 (males) and 23 (females) in 2008–2010^17^. Similarly, the WHO reported age-standardised mortality rates in 2008 were 81.4 among males and 16.9 among females^4^. The present study significantly adds to our understanding of external causes of mortality by utilising all four of the available fields in death certificates.

Mortality from external causes accounted for around 1 in 10 deaths in males and 1 in of 20 deaths among females. These ratios did not change markedly during the period under study. These ratios mirror findings from a population-based study conducted in Kenya^10^; but were lower than ratios reported in Rwanda, South Africa and Uganda^21–23^. The Kenyan study utilised data from death certificates from municipal registries, whereas the latter three countries used data extracted from hospital and police records. This contrast in the ratios of external to total mortality between countries may be partly due to very few deaths from social unrest, war or major environment cataclysms in Seychelles in the past 30 years. While there are significant social and economic differences between these countries^24^, other contextual factors may also bias the reporting of causes of external deaths reported by hospital and police sources in different countries^25^.

The ratios of external to total mortality among females and males did not change markedly during the period under study. Comparable studies examining changes in this ratio of external to total mortality are scarce in LMICs. Some studies considered only one-year periods or less^8,21–23^ or did not consider trends over time^15^. Of note, the age-standardised rates of both total mortality and mortality due to external causes decreased over time in the Seychelles, which explains why the ratio of deaths due to total vs external causes did not significantly change over time. A finer-grained analysis of the magnitude of this ratio over time is challenging as it depends on both the numerator and the denominator. The denominator (total mortality) is largely driven by causes of death other than external causes e.g. infectious diseases and non-communicable diseases (the age-standardised rates of which are decreasing in many countries, including in Seychelles). The numerator (external causes) includes causes that may be expected to either decrease (e.g. homicide, drowning, possibly due to overall better education and improving conditions of life over time) or increase over time, for example road traffic injuries. On the other hand, the relatively few numbers may have led to unstable estimates among females. The descriptive statistics are presented in Table 1. Overall, the similar ratio over time in Seychelles may be reassuring given that the age-standardised rates of both the total mortality^17^ and the mortality rates due to external causes decreased over time. The decreasing age-standardised mortality rates of external causes (and of overall mortality) in Seychelles are consistent with increasing socio-economic development over time, safer and better housing, safer working conditions, increasing education, better health and social services, and a number of social and injury prevention programs^19,26^. Relative to other African countries, Seychelles ranks highly in health, economic and population outcome indicators^14,19,26^. For example, fertility rates are quite low, life expectancy fairly high, and the burden of under-nutrition and infectious disease has become quite low^13,14,19^. With the exception of Mauritius, which has relatively similar health and socio-economic indicators^19^, this suggests that the socio-economic situation, and subsequent public health challenges may differ in Seychelles as compared to other countries within the African region.

The age-standardised mortality rates from external causes was approximately four times higher among males, which is consistent with findings in other countries, e.g. South Africa^23^. A smaller sex difference has been reported in other countries, e.g. Guinea and Malawi^8,15^. Generally, a larger incidence of external causes of deaths among males may be related to differences in risk taking behaviours and an often greater exposure to occupational injuries at work (e.g. construction workers) or at leisure time.

The three leading external causes of death were drowning, road traffic injuries and other unintentional injuries. The age-standardised mortality rates from drowning in Seychelles were highest among males aged 40–59 years (8.2 deaths per 100,000 male person-years). This is consistent with fishing being a major commercial and leisure activity in Seychelles and not all children and adults appear to be strong swimmers. Drowning was not a leading cause of mortality among children and adolescents in Seychelles, possibly because swimming in the ocean is not common and seemingly very few swimming pools exist. In comparison, drowning mortality was reported to be much higher among children and adolescents in Bangladesh, Malawi and Tanzania^11,15,27^, however, drowning has been linked with fishing activity in Malawi and Tanzania^15,28^. Programs and policies to strengthen security measures on fishing and leisure boats, and the promotion of swimming competency among children at school are important risk reduction strategies.

Road traffic injuries were the second leading external cause of mortality in Seychelles, consistent with data from Kenya and South Africa^10,23,29^. Other studies in the region have found road traffic injuries to be one of the leading contributors to mortality, especially among males^8,11,21,22,27,28^, and assault or homicide in Kenya and South Africa^10,23,29,30^. Nearly two thirds of those killed in the road traffic environment were aged 15–44 years in Seychelles, with a peak at the age of 15–29 years, as previously reported^2,6^. In Seychelles, road traffic injuries contributed 17.5% of all external deaths, which is less than observed globally at 23%, and between 37 and 46% in Guinea, Rwanda and Uganda^6,8,21,22^. The road traffic injury mortality rate per 100,000 person-years (18.0 among males and 4.6 among females in Seychelles) was within the range reported in Africa (20–31)^21,22^ and at the worldwide level (e.g. 21 in 1990 and 20 in 2013)^2^. However, mortality rates due to road traffic injuries are expected to increase over time in LMICs; from 1.2 million in 2002 to 1.9 million by 2030^31^. The increasing age-standardised mortality rates over time due to traffic injuries in Seychelles may be largely related to the dramatic increase in motorized transport (e.g. > 10 times more cars in 2018 than in 1989)^26,32^ in the context of mountainous geography, which makes it difficult to broaden roads and build physical separation for bike and pedestrian traffic. Strict enforcement of speed and drunk driving laws, building safe lanes for pedestrians and cycling, and measures to promote public versus private transport, among other interventions, have the potential to reduce road traffic injuries.

Other unintentional injuries contributed the largest number of deaths from external causes among females, particularly among those aged 60 years and above. In this study, ‘other unintentional injuries’ included hip, femur and other limb fractures for which the cause was unspecified and can include injuries consecutive to simple falls. Falls are a major external cause of mortality in many countries, especially among the elderly^33,34^. In light of this, falls among the elderly may therefore be under-estimated in this study. Other unintentional injuries in Seychelles included electrocution, shark attacks, aircraft accidents, ship injuries, rock accidents, falling objects, lightning, asphyxiation, and injuries due to epileptic seizures, which mostly occurred among the younger age groups. The higher proportion of other unintentional injuries in females than in males may be partly due to the fact that life expectancy at birth is 8 years higher in females than in males in Seychelles (e.g. males 70.4 years, females 78.4 years in 2017). This emphasises the increasing need to address pathologies due to frailty in the aging population, particularly in relation to the prevention and management of falls among the elderly.

## Strengths and limitations

The strengths of this study include that all deaths occurring in the entire population during the last three decades were included and all of them were medically certified. All the external causes of death were reviewed and recoded for this study; and the whole population was enumerated. This facilitated a reliable comparison of the externally caused age-standardised mortality rates during the considered thirty-year period. Additional analyses for the top four categories were conducted for 5-year periods and 15-year periods (Table 5). These estimates did not change the interpretations derived from the 10-year periods. Furthermore, undercounting was unlikely in these data because the registration of deaths was centralised and covered the entire country. The study also has limitations. Because the country population is small, the numbers of deaths are also small, and mortality estimates for some specific external causes were unstable over time. Also, the accuracy of the causes of death written in the death certificates by the certifying physicians may not always be optimal as physicians are not necessarily sufficiently trained in reporting causes of death along the ICD classification. This imprecision is reflected by the fairly high proportions of deaths from unspecified external causes. Some external causes of death are also inherently difficult to assess accurately. For example, suicide may be underreported due to unclear circumstances of death, e.g. accidental versus voluntary poisoning. Additionally, comparisons between all external cause categories could not be analysed in depth since causes of death may not always be allocated precisely. For example, drowning may result from an unintentional fall (e.g. from a boat) into a body of water or be intentional (suicide) or caused by someone else (homicide). Given that a complete social enquiry on the circumstances around a death is not necessarily done, it may not be possible to elucidate the definite category of external causes for a number of recorded deaths, preventing further precise analyses. Furthermore, drug abuse, and related fatal complications, can be difficult to assess at the time of death. In some studies, drug abuse has directly or indirectly contributed to nearly half of all drug addict fatalities^35^ and a longer duration of drug use has been associated with an even higher mortality^36,37^. However, this study included cases linked to drug abuse and addiction. Finally, the situation of Seychelles, a rapidly developing, peaceful, small island state, limits the generalizability of the findings to other countries in the African region but results may possibly be partially inferred to other rapidly developing small island states in different regions.

**Table 5:** Crude and age-standardised mortality rates by 15- and 5-year intervals for the top 4 categories of external causes of death.

	Crude mortality rate		Age standardised mortality rate		95% Confidence interval	
	M	F	M	F	M	F
**External causes**						
Every 15 years						
1989–2003	107.2	26.2	120.0	25.8	110.2, 129.0	21.6, 30.0
2004–2018	97.5	30.0	96.1	25.8	88.7, 103.6	22.1, 29.4
Every 5 years						
1989–1993	106.4	29.1	130.9	30.6	111.1, 150.7	21.8, 39.3
1994–1998	113.7	25.6	125.4	24.0	108.1, 142.7	17.1, 30.8
1999–2003	101.8	24.3	107.3	23.8	92.2, 122.4	16.9, 30.6
2004–2008	92.5	25.8	96.2	23.0	82.4, 109.9	16.7, 29.3
2009–2013	93.6	35.3	94.0	29.5	81.0, 107.1	22.8, 36.2
2014–2018	105.7	28.6	102.3	25.3	89.1, 115.4	18.8, 31.8
**Drowning**						
Every 15 years						
1989–2003	27.2	2.6	30.8	2.9	25.8, 35.8	1.4, 4.4
2004–2018	23.2	4.1	22.8	4.0	19.2, 26.4	2.5, 5.5
Every 5 years						
1989–1993	29.5	4.6	37.1	4.9	26.5, 47.6	1.4, 8.4
1994–1998	28.4	1.6	31.4	1.3	22.7, 40.0	0.1, 2.9
1999–2003	24.1	1.9	26.2	2.5	18.6, 33.8	0.1, 5.0
2004–2008	19.7	3.8	21.3	3.9	14.7, 28.0	1.2, 6.7
2009–2013	22.1	1.8	22.5	1.6	16.1, 28.9	0.1, 3.3
2014–2018	27.5	6.4	24.6	5.9	18.4, 30.7	2.8, 9.0
**Road traffic injuries**						
Every 15 years						
1989–2003	16.1	4.7	16.4	5.0	12.9, 19.9	3.1, 7.0
2004–2018	20.0	4.5	19.5	4.5	16.2, 22.9	2.9, 6.2
Every 5 years						
1989–1993	14.5	4.0	15.1	4.8	8.9, 21.3	1.1, 8.4
1994–1998	17.4	3.7	18.1	4.2	11.7, 24.5	1.0, 7.4
1999–2003	16.2	6.3	16.0	6.2	10.4, 21.6	2.8, 9.6
2004–2008	18.8	4.3	18.6	4.3	12.7, 24.5	1.5, 7.2
2009–2013	18.5	5.4	18.0	5.1	12.4, 23.6	2.2, 8.1
2014–2018	22.4	3.8	24.2	4.4	17.4, 30.9	1.3, 7.5
**Other unintentional injuries**						
Every 15 years						
1989–2003	13.8	9.4	17.1	8.2	13.1, 21.0	6.0, 10.4
2004–2018	10.7	11.3	12.2	7.7	9.3, 15.1	5.9, 9.5
Every 5 years						
1989–1993	15.6	9.1	20.5	8.2	12.3, 28.7	4.1, 12.2
1994–1998	12.1	11.5	15.2	10.2	8.8, 21.6	5.9, 14.5
1999–2003	13.8	7.8	15.6	6.5	9.6, 21.6	3.2, 9.8
2004–2008	9.9	10.0	11.0	7.3	6.2, 15.9	4.1, 10.5
2009–2013	9.9	16.3	11.6	11.5	6.6, 16.6	7.6, 15.4
2014–2018	12.3	7.7	13.5	5.0	8.4, 18.6	2.6, 7.4
**External causes, undetermined**						
Every 15 years						
1989–2003	20.1	3.1	23.5	3.3	19.1, 28.0	1.7, 4.8
2004–2018	8.6	2.1	8.4	1.8	6.2, 10.6	0.8, 2.8
Every 5 years						
1989–1993	24.3	4.6	30.2	5.4	20.5, 39.9	1.5, 9.2
1994–1998	18.4	3.1	20.6	2.8	13.6, 27.6	0.5, 5.0
1999–2003	18.2	1.9	20.6	2.2	13.8, 27.4	0.1, 4.5
2004–2008	8.5	1.9	9.4	1.7	4.9, 13.9	0.1, 3.4
2009–2013	7.2	1.8	6.3	1.6	3.2, 9.5	0.1, 3.3
2014–2018	10.1	2.6	9.8	1.8	5.7, 9.8	0.3, 3.3

*M* males; *F* females.

## Conclusions

External causes contributed around to 1 out of 10 deaths in males and 1 in 20 deaths in females in Seychelles, and these proportions did not change markedly over time. Considering that drowning and road traffic injuries were the leading external causes of mortality, measures to address speed and drunk driving, to promote road safety and to improve security on board boats and near water bodies, are likely to minimise externally caused mortality. These data also emphasise the need for reliable vital statistics at the entire population level, and the great potential of such data to guide prevention programs and policy in multiple sectors.
